# The Wine Industry By-Products: Applications for Food Industry and Health Benefits

**DOI:** 10.3390/antiox11102025

**Published:** 2022-10-14

**Authors:** Raúl Ferrer-Gallego, Paula Silva

**Affiliations:** 1 Centro Tecnológico del Vino (VITEC), Ctra. Porrera Km. 1, 43730 Falset, Tarragona, Spain; 2 Bodega Ferrer Gallego, 46311 Jaraguas, Valencia, Spain; 3 Department of Ecology, Desertification Research Centre (CIDE-CSIC-UV-GV), 46113 Moncada, Valencia, Spain; 4 Laboratory of Histology and Embryology, Institute of Biomedical Sciences Abel Salazar (ICBAS), Rua de Jorge Viterbo Ferreira n° 228, 4050-313 Porto, Portugal; 5 iNOVA Media Lab, ICNOVA-NOVA Institute of Communication, NOVA School of Social Sciences and Humanities, Universidade NOVA de Lisboa, 1069-061 Lisbon, Portugal

**Keywords:** grape pomace, circular economy, Mediterranean diet, wine by-products, polyphenols antioxidants

## Abstract

Each year, 20 million tons of wine by-products are generated, corresponding to 30% of the total quantity of vinified grapes. Wine by-products are a source of healthy bioactive molecules, such as polyphenols and other molecules (pigments, fibers, minerals, etc.). The abundance of bioactive compounds assures a promising future for nutritional foodstuff production. Wine by-products can be used to fortify aromatized waters and infusions, bread, pasta, dairy products, alcohol, sugary beverages, and processed foods. These innovative products are part of the Mediterranean diet and are of great interest to both human and environmental health. Pre-clinical studies show that consumption of food produced with wine by-products or with their extracts attenuates the inflammatory state and increases antioxidant status. As such, wine by-products provide protective effects against the underlying pathophysiological hallmarks of some chronic diseases such as atherosclerosis, diabetes, hypertension, obesity, and cancer. However, the poor bioavailability warrants further investigation on how to optimize the efficacy of wine by-products, and more clinical trials are also needed. The scientific evidence has validated the uses of the dietary nature of wine by-products and has helped to promote their use as a functional food to prevent chronic human diseases.

## 1. Introduction

The total global production of wine in 2021 was estimated at around 250 million hectoliters [1]. The 30% of the total quantity of vinified grapes corresponds to wine by-products that represents nearly 20 million tons, of which 50% corresponds to the European Union. Wine by-products have been used for different purposes, in agriculture, cosmetics, pharmacy, biorefinery, feed and for food industry [2,3,4,5,6,7,8]. The main waste streams with food interest are grape pomace (GP) and wine lees [9]. GP is the residue originated after the pressing of red and white grapes to produce must or wine. It is composed of stems, skins, and seeds. During the vinification process, a large amount of GP is generated with valuable recovery because it constitutes an important source of value-added products such as phenolic compounds, mainly flavonoids, phenolic acids, and stilbenes [10,11]. The solid deposits formed at the bottom of the tanks mainly consist of yeasts and bacteria, carbohydrates, polyphenols, lignin, proteins, metals, salts, and organic acids (e.g., tartrates). The liquid phase is rich in ethanol and organic acids (tartaric acid, lactic acid, and acetic acid) and is usually distilled to recover ethanol and produce distilled beverages [12]. The clusters or grape stems constitute a residue that can be used as a source of astringent compounds, represented mainly by proanthocyanins. Wine lees also have important antioxidant and biological properties [13]. All these compounds should be reused for food industry and the enrichment of products included in the Mediterranean diet (MD). It is well known that MD has a low environmental impact, since it is mainly a plant-based diet with low consumption of animal products, a small water footprint and low greenhouse gas emissions [14]. Removing wine industry by-products is a suitable strategy for recovering bioactive compounds (mainly polyphenols) from GP and reducing the environmental impact of these industrial wastes.

MD is based on the traditional foods eaten by people living in the Mediterranean region, such as fruits, vegetables, beans, nuts, seafood, olive oil, and dairy products with perhaps a glass of wine, and inversely associated with consumption of ultra-processed foods and lower free sugar intake [15]. Consumers are increasingly looking for solutions to improve health and prevent chronic diseases. This is one reason why everyone is talking about MD nowadays. In fact, according to U.S. News & World Report 2020, MD is the number one overall diet for the third consecutive year. The main reason for this success is the great amount of evidence achieved by scientists in showing that MD is one of the healthiest dietary patterns, with several benefits regarding longevity and chronic diseases [15]. Recently, MD has been promoted as a useful tool to reduce body weight, which could also explain the current interest in this diet. However, it remains uncertain what consumers understand about and expect from MD. Knowing that consumers quickly quit diets because the effort required is perceived as outweighing the benefits, it is important that physicians, nutritional authorities, scientists, educators, cooks, and communicators work together to convince consumers that MD is not just something that can be done for a short time to be healthier [16] and/or to lose weight, but it is a lifestyle that must be adopted forever.

**Figure 1:**
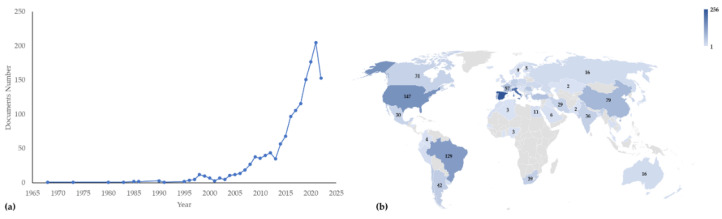
Number of documents with “grape pomace” in the title, abstract and keywords by year (**a**) and by country (**b**) according to Scopus on 19 September 2022 (Total number of documents = 1473).

The use of GP is relatively recent, in fact, most papers were published in the last decade (Figure 1a). The sources of literature that include peer reviewed journals show a high increment of articles related to GP from 2015 to now. This trend notices the importance of the revalorization of wine by-products in the last years. Regarding countries, Spain, Italy, the United States, Brazil, Portugal, China, and France are responsible for more than a half of scientific production (Figure 1b).

The global wine industry has been constantly growing over the last decades. However, the wine industry results in significant amounts of by-products, which have negative consequences for the environment. This practice is not acceptable and must be stopped. The urgency for sustainability within the wine industry has turned research interests to investigate the management of wine by-products from another perspective, for example, by adapting more profitable options and using wine by-products to produce foods. The current review is of the utmost importance and aims to indicate alternative solutions for the upgrading of wine by-product processing, as well as indicating their industrial potential in the food industry. The goal is to support the scientists, professionals, and enterprises that aspire to develop high-scale industrial applications. It focuses on the most recent advances in the area, while also analyzing the potential of already produced foods and their potential health benefits. The review fills an existing gap in the current literature by providing a direction for all the involved stakeholders, professionals, and technologists who are active in the field and are trying to optimize the wine industry’s performance and reduce its environmental impact. The optimization of wine waste management, the improvement of enriched foods and drinks, the verification of some related health benefits of polyphenols, and finally its inclusion on Mediterranean markets leads the purpose of this review. The objective is to review the health benefits of bioactive compounds found in winemaking by-products and their application to produce innovative food products that can be included in MD. Sustainable use of the enrichment of MD products from GP to promote new food attributes including health and nutritional benefits, environmentally friendly food, and socially responsible food is reported here.

## 2. Food Industry Applications

### 2.1. Food Pyramid of Wine-By Products

The enrichment of foods with winemaking by-products will create functional food items and can allow for the introduction of natural functional food ingredients (mainly dietary fibers, and polyphenols) in commonly consumed foods. A reduction in environmental impact is also a derived benefit of these practices. Redesigning common MD foods and creating contemporary products that could have a great acceptability by consumers can increase the competitiveness of the food industry, as well as guide consumers, especially the younger ones, towards more sustainable and healthy choices.

**Figure 2:**
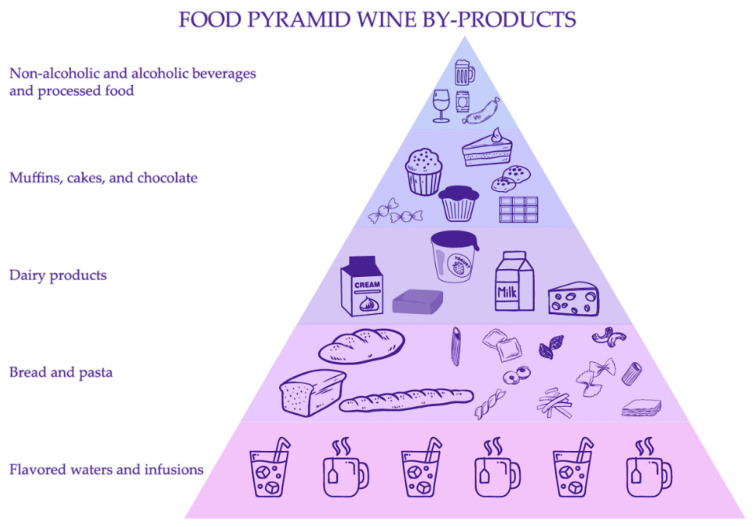
Valuable applications of wine by-products in the food pyramid of the Mediterranean Diet.

The food guide pyramid is a recognizable nutrition tool that was introduced by the United States Department of Agriculture (USDA) in 1992. It was shaped like a pyramid to suggest that a person should eat more foods from the bottom of the pyramid and fewer foods and beverages from the top. The food pyramid of MD based on wine by-product enrichment could be constituted on five groups of foods and beverages (Figure 2). From the base to the top, we find aromatized waters and infusions, breads, pasta, dairy products, alcohol, sugary beverages, and processed foods. All of them can be fortified by adding GP extracts derived from wine industry wastes to produce innovative products that could be included in MD with some interest in our health, and in our planet. GP is a winery by-product that is more and more valorized as a source of healthy bioactive molecules, such as polyphenols and other interesting molecules (pigments, fibers, minerals, etc.) [17,18].

### 2.2. Flavored Waters and Infusions

According to the last Market Data Forecast report, the global flavored water market size was calculated at USD 17.2 billion in 2021 and is estimated to grow up to USD 9.69 billion by 2026 [19]. Flavored waters are at the base of Food Pyramid Wine-by products (Figure 2). They belong to the category of beverages that exclude ingredients, such as artificial flavors, vitamins, sweeteners, or others. Flavored and colored water could be bottled water, purified water, or spring water, as derivative water with added functional value. Increasing consumer demand for natural flavors and fragrances has driven up prices and increased pressure on natural resources. The food industry is trying to popularize wine-like drinks that boast colors and ingredients derived from red grape waste. Color positively influences the consumer’s preference, purchase decision and eating desires, playing a noticeable role in the acceptability of these beverages. Recently, there has been a worldwide movement towards increased use of natural colorants [20,21,22,23]. GP from red grapes is a great source of natural pigment, which contains anthocyanins, mainly 3-glycosides, 3-acetylglycosides; and 3-p-coumaroylglycosides of malvidin, peonidin, delphinidin, petunidin and cyanidin [24].

In the same way as color, volatiles of GP have interesting flavors that can be used to improve the aromatic profile of some waters and fruit teas. The extraction of aromas from GP has been studied along time [25,26,27,28]. In 1993, Vasserot and co-workers [27] reported the aromatic profile of Muscat GP. Ruberto et al. [28]) reported the volatile components of GP from different Italian grape varieties. Muñoz-González et al. [26] identified a total of 22 varietal aroma compounds from Verdejo GP, the most represented compounds being terpenes and volatile phenols. Liang et al. [25] reported 65 volatile compounds from Chardonnay GP, in this case considering alcohols and volatile phenols the most abundant ones. Many of the volatile compounds found in GP have pleasant aromatic notes related to herbal, fruity, or floral perception [25]. Recently, Lindsay and co-workers [29] reported that some filamentous fungi find grape marc could be valuable sources of aromas. *Aspergillus niger* and *Aspergillus oryzae* are able to produce metabolites also associated with pleasant flavors, such as herbal notes, citrus (3-octanol), lavender, nectarine (3-octanone), cinnamon (E-cinnemaldehyde), or tropical fruits (ethyl tiglate) [29]. The popularity of fruit teas is increasing in the world because of their antioxidant properties and their attractive taste. The addition of natural aromas recovered from GP could be a great option for further improving demand.

The bioactivity of GP has also been evaluated in fortified waters and infusions. Recently, Costa et al. [30] reported a decrease in the growth rate of pathogens after gastrointestinal digestion in coconut water fortified with encapsulated GP extract. GP is not only a rich source of polyphenols, but also shows enzyme activities, antimicrobial effects, antioxidant, and anti-inflammatory activity with interest in producing functional drinks [31,32,33,34].

### 2.3. Bread and Pasta

The addition of GP powder to substitute flour for wheat bread fortification has been used to enhance the bioactive potential and physical properties of bread and pasta [35,36]. In this way, Monteiro et al. [37] used different Brazilian grapevine hybrids to enrich flours for potential use in the food industry. Antioxidant activity and consumer acceptance have also also evaluated in cereal bars and bakery products with differing flour content made with Merlot and Cabernet Sauvignon GP [38]. The addition of Cabernet Sauvignon GP powder may also improve the rheological and microstructural properties of wheat dough [39].

In the case of pasta made with GP-enriched flour, fortified tagliatelle retained anthocyanins and hydroxytyrosol, and showed good texture after cooking. Moreover, tagliatelle fortified with GP enhances the nutritional profile and increases fiber content [40]. Fettuccini and spaghetti based on grape marc powder increased their polyphenol content and antioxidant activity, resulting in an interesting alternative regarding final product costs [41,42].

### 2.4. Dairy Products

Yoghurt has gained positive perception by consumers as a functional dairy product with health-promoting ingredients. Yoghurt with added antioxidants from GP appears to be a convenient food format for satisfying consumer interest [43]. In this way, wine by-products can not only improve their functionality, but also their sensory attributes such as texture, creaminess, color, and aroma [44]. Moreover, the addition of GP may be used to improve the storability of dairy products, extending their shelf life [45]. It has been demonstrated that the application of GP may substantially increase the total phenolic content and antioxidant activity, which are not present in dairy products [46]. GP powder from Chardonnay was used to increase polyphenols in semi-hard and hard cheeses. Results showed that particular attention is needed concerning effects on pH that may increase acidity depending on the type of the extract added. However, GP can be a functional ingredient for increasing the total phenolic compounds and radical scavenging activity of cheese [47]. From a microbiological point of view, the ability of the starter strains to drive the fermentation process in the presence of GP has also been evaluated, showing good results in many cases [47,48]. Furthermore, the addition of GP can reduce the fat content of cheese and increase protein levels and secondary lipid oxidation [47]. GP can serve as coagulant in the production of tofu and may change its textural parameters, and even its color [49]. In summary, several studies have revealed that wine by-products are relevant as a source of functional compounds for the formulation of dairy products, such as fermented milks, yogurts, cheeses, or ice-creams [50,51,52].

### 2.5. Muffins, Cakes and Chocolate

At the top of the pyramid, we find foods for occasional consumption. Muffins, cookies, and sugary products have also been an object of study for incorporating GP. Hence, Aksoylu and co-workers [53] showed that the addition of GP powder to biscuits decreases free fatty acids and increases protein content. In this study, the sensory acceptability of biscuits reached five months of storage [53]. It is well reported that GP can be an important source of fiber and polyphenols, which increase the functional properties of biscuits and sweet cookies [54,55,56,57]. Furthermore, GP additions may be an alternative to gluten-free products for improving their nutritive and healthy properties [58]. There are different methods for adding GP extracts to sweets. Thus, Spigno and co-workers [59] reported that encapsulated freeze-dried GP powder may help preserve hazelnut paste in the oxidation process during the storage period; the resulting oil/water nanoemulsion being the best system for it [59]. In chocolate, the use of GP as an additive may also improve its rheological properties and digestibility [60,61].

### 2.6. Non-Alcoholic and Alcoholic Beverages and Processed Food

The effect of winery by-product extracts on oxidative stability, volatile organic compounds, and the aroma profile of beverages (with and without alcohol) and processed food was also investigated in the last years.

Regarding non-alcoholic and alcoholic beverages, very few studies were carried out to explore the potential use of wine industry by-products in their production. Recently, the incorporation of GP extracts encapsulated alginate or chitosan microparticles to produce a functional coconut beverage for the first time. This incorporation did not affect most coconut water sensory attributes, including aroma and flavor. Gastrointestinal digestion does not affect most of the sensory attributes of coconut water, and bioactive molecules reach the intestine with minimal losses. Storage at 4 °C allowed for a reduced degradation rate of total phenolics and anthocyanins of the fortified beverage. The potential antimicrobial activity of digested functional coconut water beverages against intestinal pathogens was validated and related to the high level of total phenolics and total anthocyanins [30]. Despite the controversial health effects of wine and beer, these beverages can also be improved with the addition of wine by-products. The addition of grape seeds obtained from GP (Pedro Ximénez variety) increases the flavonoid content and consequently the antioxidant activity in red wines in a warm climate. More studies are needed to elucidate the components responsible for the increase in antioxidant activity. However, these results are of great interest to the wine industry [62]. The addition of white GP (both pasteurized and unpasteurized) of the Solaris variety increases the concentration of many volatile components of beer, such as ethyl decanoate or ethyl dodecanoate, influencing sensory characteristics. Beers produced with white GP have higher concentrations of phenolic compounds, as well as antioxidant properties. Moreover, the authors showed that beers produced with white GP have a lower amount of acetaldehyde, which is a toxic compound. With the addition of fruit, the concentration of sugars in the beer decreased while the concentration of organic acids such as tartaric, malic, and succinic increased [63].

It is well known that wine industry by-products (specifically wine pomace) can be used to extend the shelf life of foodstuffs by preventing oxidative degradation and controlling the growth of spoiler microorganisms [64]. Moreover, considering the restrictions imposed through legislative regulations set by the European Commission, these by-products seem to be safe, as was already proved for grape flour [18]. GP phenolics (0.1 g/kg tissues) reduce the concentration of essential fatty acids (PUFAs), eicosapentaenoic, and docosahexaenoic acid and, consequently, were effective in preventing lipid oxidation in fish tissues, which leads to product quality improvement [65]. A different study revealed that grape-seed phenolic extract is much more efficient in fish tissues compared to GP, with nearly 1.3 g of phenolics per kg of fish tissues [65]. Also, the addition of grape antioxidant dietary fiber in chicken hamburger and fish muscles was effective in scavenging free radicals and improving oxygen stability [10]. The addition of nano-emulsion-based capsules produced from GP phenols to hazelnut paste inhibits undesired oxidation [66].

Despite the above-mentioned advantages, the application of these products to produce fortified processed food is in its early stages. The addition of 0.5% grape seed pomace in pork sausages retards lipid oxidation; however, 1% grape seed pomace did not effectively scavenge ROS formation due to the confirmation of TBARS. Therefore, grape seed pomace could trigger antioxidant activities in cooked pork sausages [67]. Recently, Tremlova et al. [68] carried out a study to experimentally produce vegetarian sausages without the application of heat treatment and with the use of grape flour. They evaluated the influence of the addition of grape flour on the chemical, physical, and sensory properties of this vegan sausage. Grape flour addition to vegan sausages increased antioxidant capacity and increased polyphenol content [68].

## 3. Health Benefits

**Figure 3:**
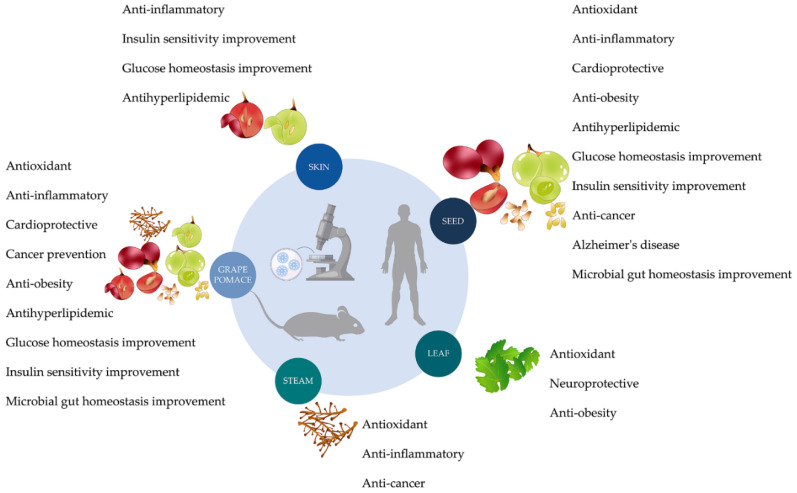
Wine by-product bioactivity showed by pre-clinical and clinical trials.

Wine by-products used to enhance foods, such as baked goods, pastries, and pastas, improve the product’s nutritional and functional value by increasing fiber and antioxidant compounds [69]. The remaining question is whether that improvement has health benefits. In this section, we will summarize the results of in vitro and in vivo experiments and clinical trials performed to clarify the potential health effects of wine by-products used in the food industry (Figure 3).

In this section, we will summarize the potential health benefits of foods produced with wine by-products or with their extracts. However, the health benefits of wine by-products can also be speculated by looking for phenolics recovered from them. The amount and type of phenolic compounds present in wine by-products depend on the cultivar, climatic conditions, and extraction technique applied. Usually, they are rich in phenolic acids, flavonols, proanthocyanidins, anthocyanins, and stilbenes. The phenolic composition of grape pomace is presented in Table 1; discussion of the health benefits of isolated phenolic compounds is not within the scope of this review.

### 3.1. Grape Pomace

When thinking about the health benefits of food enriched with wine by-products, it is reasonable to assume that gut microbiota could be one of the first targets. These products are rich in polyphenols that exert prebiotic-like effects by stimulating the growth of beneficial bacteria and inhibiting pathogenic ones [88]. It was found that GP extract (a mixture of Cabernet Sauvignon, Marselan, and Syrah varieties) improves microbial gut homeostasis of rats fed with it for 14 months (2.5–20.0 mg polyphenolics/kg body weight/day). This GP extract was able to inhibit non-beneficial bacteria from the rat microbiota and potentiate the growth of probiotic ones [89] (Figure 3, Table 2). GP also makes a difference in gut microbiota modulation during a pathological situation. For example, in pigs infected with *Ascaris suum*, administration of GP appeared to modulate gut function by regulating prokaryotic gut microbiota composition and/or changing colonic short-chain fatty acid concentrations in the colon. Moreover, GP extract improved the immune response with an increase in the number of eosinophil and mast cells in pig intestine mucosal [90]. Supplementation with GP also improves the microbial composition of C57BL/6J mice fed a high-fat diet (HFD) for eight weeks. In GP-treated mice, a decrease of *Desulfovibrio, Lactococcus*, and an increase of *Allobaculum* and *Roseburia* was observed. Moreover, the expression of several antimicrobial peptides and tight junction proteins increases in response to GP supplementation, suggesting an improvement of mouse gut barrier function (Figure 3, Table 2). The GP extract also promoted a reduction of fat mass gain and adipose tissue inflammation in HFD-mice, which could be associated with reduced liver steatosis and lower plasma non-esterified fatty acid levels (Figure 3, Table 2). GP had a beneficial effect on glucose homeostasis, by improving glucose tolerance and lowering the insulin resistance index (Figure 3, Table 2). Together, these data suggest that GP ameliorates gut microbiota, which is partly responsible for the overall metabolic profile improvement of mice on an HFD [91]. Similarly, GP supplementation influences the recovery of gut microbiota after antibiotics and HFD treatment [92]. Despite the promissory results from in vivo studies, results from clinical trials are still controversial. A randomized crossover clinical trial with freeze-dried and milled GP (8 g day^−1^, 6 weeks) suggests that the reduction of insulin levels observed in overweight subjects at cardiometabolic risk by GP is not induced by changes in gut microbiota [93]. Another clinical trial found a relationship between health and the gut microbiome in subjects who consumed 2 g day^−1^ of the GP-derived seasoning, as a sodium salt replacement in their foods, for six weeks [94]. However, the human response to polyphenol supplementation is not homogeneous. The variations in fecal microbiota and miRNA expression may be valuable when explaining this inter-individual variability in clinical trials with GP. A clinical trial with daily supplementation with 8 g of dried GP for six weeks suggests both gut microbiota and miRNAs can be indicators of insulin responsiveness to GP among obese subjects at high cardiometabolic risk. The individuals with higher levels of plasma insulin concentration were sensitive to GP (responders) while showing reduced levels of *Firmicutes* and *Prevotella*, together with increased expression of miR-222 [95]. Animal studies show that GP have the potential to modulate gut microbiota composition, by changing the growth of different microbial species and/or changing the community dynamics, modulating the microbial population of the colon considerably. However, clinical studies with GP have controversial results. Therefore, in the future, more dietary intervention studies are needed to fully understand the impact of GP on health, and to understand the strengthened causal connections between dietary aspects and health-related microbial modulation.

Besides gut microbiota, GP benefits were already tested, by Calabriso et al. [96], for their potential to diminish the overwhelming inflammatory response in enterocyte-like cells. They carried out an in vitro study with intestinal epithelial Caco-2 cells, grown in monolayers or in co-culture with endothelial cells (Caco-2/HMEC-1) [96]. Cultures were treated for two hours with different concentrations of GP (1, 5, 10 µg/mL gallic acid equivalents) and then stimulated, for 16 h, with lipopolysaccharide (LPS) and tumor necrosis factor (TNF). They found that GP supplementation prevents, in a concentration-dependent manner, the intestinal expression and release of interleukin (IL)-6, monocyte chemoattractant protein (MCP)-1, and matrix metalloproteinases (MMP)-9 and MMP-2. Moreover, in Caco-2 cells, GP suppressed the gene expression of several proinflammatory markers, such as IL-1, TNF, macrophage colony-stimulating factor, C-X-C motif ligand-10 (CXCL10), intercellular adhesion molecule (ICAM)-1, vascular cell adhesion molecule (VCAM)-1, and cyclooxygenase-2 (COX-2). The authors showed that both NF-κB (nuclear factor kappa light chain enhancer of activated B cells) activity inhibition and intracellular reactive oxygen species (ROS) levels reduction mediated the GP anti-inflammatory effect. Regarding endothelial cells, the GP supplementation decreased the endothelial expression of IL-6, MCP-1, VCAM-1, and ICAM-1. Because of that, GP inhibited the adhesion of leukocytes to the endothelial cells under pro-inflammatory conditions [96]. This ability of GP to modulate various pathways involved in inflammation and oxidative stress observed in vitro could help explain the protection previously observed in rats. The oral administration of GP at 0.1% for 21 days, followed by acute colitis induced by dextran sulfate sodium (40 g/L in the drinking water) administration over the last 7 days resulted in a delay of the onset of colitis symptoms and prevented a decrease in food intake, animal weight loss, colon shortening, and the polymorphonuclear infiltration of the intestinal wall [97]. These data show that GP can promote the secretion of anti-inflammatory factors by improving the intestinal mucosal barrier and immune system function. It seems that through the NF-κB signaling pathway, GP decreases proinflammatory cytokines such as IL-6 and TNF. On the other hand, GP seems to alleviate oxidative stress and decrease the risk of inflammation. Further studies, in suitable models, are needed to confirm these hypotheses and validate potential modalities for the GP prevention of intestinal inflammation. Moreover, considering the effects of GP on intestinal microbiota, GP potential to reduce intestinal infections through intestinal microbiota regularization should be investigated in the future. These results also open avenues for exploring the effect of GP gut chronic inflammatory diseases and for all diseases in which the improvement of vascular endothelial function is important.

Cardiovascular protection is one of wine polyphenols’ best-known. Therefore, it is reasonable to expect that GP could also have these effects. Diahann Perdicaro and co-authors [98] observed that GP (*Vitis vinifera* L. Malbec variety, Argentina) supplementation prevented the appearance of arrhythmias in high-fat-fructose-fed Wistar rats, mainly due to a preventive effect against nitric oxide (NO) release and bioavailability [98]. Another in vivo experiment with male Wistar rats (200–250 g) was carried out by Balea et al. [99] to test the potential in vivo cardioprotective effect of fresh (high condensed tannins and anthocyanin contents) and fermented (high polyphenolic content) GP extracts from *Vitis vinifera* L. Pinot noir cultivar. The authors found that GP treatment effectively attenuates myocardial infarction in rats by reducing serum oxidative biomarkers, such as malondialdehyde (MDA), and increasing serum total oxidative status and antioxidant reserves [99] (Figure 3, Table 2). Six different extracts from GP, selected by their antioxidant activity, were studied in vivo for six weeks with spontaneously hypertensive rats. The results showed that it is feasible to modulate anti-hypertensive effects by amplifying or decreasing polyphenol absorption [100]. The determination of the specific mechanism, the best concentrations, and ideal treatment methods require further study before clinical trials to test the GP cardioprotective effects could be conducted.

The effect of diet supplementation with GP and GP extract (GPE) on insulin-sensitive tissues (adipose, liver and, muscle) was evaluated with an experimental model of metabolic syndrome (MetS). This animal model was induced by giving a high-fat-fructose (HFF) diet to Wistar rats for six weeks. At the end of the trial, HFF diet-induced weight gain was partially attenuated by GP and GPE supplementation. HFF diet increased systolic blood pressure (SBP), triglycerides, insulin resistance, and inflammation (c-reactive protein (CRP)) (Figure 3, Table 2). Supplementation with GP prevented SBP, triglycerides, and CRP increase, and partially attenuated insulin resistance. Regarding GPE, the results showed a partial SBP and triglyceride reduction and insulin resistance and inflammation prevention. Supplementation, mainly with GP than with GPE, attenuated liver triglyceride content and adiposity and restored adipose, liver, and muscle response to insulin. Together, these results show that GP supplementation can counteract adiposity, inflammation, liver damage, and impaired insulin signaling associated with MetS [101] (Figure 3, Table 2). GP supplementation also could have beneficial effects on diabetes prevention, as shown in a study with streptozotocin (1 × 150 mg/kg)-treated mice fed with HFD supplemented with 2.4 g/kg GP for 12 weeks. In this study, a decrease in blood glucose and a hemoglobin A1C improvement was observed. Moreover, GP treatment down-regulated the expression of several cytokines involved in chronic low-grade inflammation triggered by a high-fat diet, and attenuated insulin resistance in HFD-induced diabetic mice [102] (Figure 3, Table 2). The potential of GP supplementation (50–250 mg/day) for the treatment of non-alcoholic fatty liver disease (NAFLD) was investigated using C57Bl/6 mice HFD or western diet (WD) as models of obesity and hepatic steatosis or steatohepatitis, respectively. It was observed that GP slows the progression of the disease toward this advanced liver pathology. However, GP was ineffective in reversing hepatic damage in advanced NAFLD in WD-fed mice. Moreover, in obese mice, it was observed that GP improves the metabolic profile, as indicated by reduced body weight, improved glucose tolerance and insulin sensitivity, better lipid profile, and lack of adipose tissue inflammation and ectopic fat deposition. According to the results, the authors suggested that an increase in insulin sensitivity is the main mechanism mediating the improvement of the metabolic profile observed in pomace-treated obese mice [103]. In summary, GP can improve disrupted glucose homeostasis in the insulin resistance state and has hypolipidemic effects. The precise mechanisms underlining these beneficial effects of GP are not yet entirely understood, but are probably associated with its anti-inflammation proprieties. These studies are very important, since the need to fight the metabolic disease epidemy that we are facing is urgent. It is also very important to try to achieve this in a sustainable way, using food produced with by-products such as those from the wine industry.

GP effects were also investigated in fish. A recent study was carried out to evaluate the effects of the inclusion of GP flour (GPF) on the diet of *Labeo rohita* infected with *Flavobacterium columnaris*. Harikrishnan et al. [104] found that growth rate, hematology, and biochemical parameters increased when fed with 200 and 300 mg GPF enriched diets in both normal and challenged fish. Moreover, the authors reported that the activities of antioxidants and innate-adaptive immune parameters such as MDA, superoxide dismutase, glutathione peroxidase, glutathione, phagocytic, respiratory burst, alternative pathway complement, lysozyme, and total immunoglobulin M increased in both groups. Moreover, an increase in the expression of the immune, antioxidant, and anti-inflammatory-related gene mRNA expression was observed in head kidney tissues. According to the results, co-authors concluded that, both in normal and challenged fish, supplementation with 200 mg GP flour promoted the best results regarding growth rate, antioxidant status, and immune defense mechanisms *Labeo rohita* against the gram-negative rod bacterium of the genus *Flavobacterium* [104].

In vitro studies also show GP’s potential to prevent cancer [105,106]. Again, GP antioxidant proprieties are the ones that justify the obstruction of the occurrence of cell malignancy. The attenuation of ROS and reactive nitrogen species could result in irreversible DNA damage and/or inhibition of cancer cell proliferation. The anti-proliferative and anti-genotoxic effects against colon cancer cells of red wine pomace (*Vitis vinifera* cv. Tempranillo variety, Spain) seasonings could be related to hydroxybenzoic acids and hydroxycinnamic acids [105].

### 3.2. Skins

As previously mentioned, grape skin can be also used as a functional ingredient in the formulation of food, and it was what Adriana Maite Fernández-Fernández and their colleagues (2022) did when they produced yogurt and biscuits [107]. An analysis of the snacks revealed that the phenolic portion was mainly composed of flavonoids, phenolic acids, and anthocyanins. Moreover, the authors simulated oral gastrointestinal conditions to obtain grape skin bioactive compounds and then estimate their bioaccessibility and health-promoting properties (antioxidant, anti-inflammatory, and antidiabetic). They found that GP addition increased the 2,2′-azino-bis(3-ethylbenzothiazoline-6-sulfonic acid) antioxidant capacity of the biscuits and increased the glucosidase inhibition capacity of the yogurt. Interestingly, the digested yogurts presented more antioxidant capacity than the digested biscuits. According to the authors, these results are related to yogurt bioactive peptide release during digestion and are probably due to a possible interaction between grape seed polyphenols with macromolecules from the snack’s ingredients, which can affect their biological effects. Moreover, the enriched yogurt reduced ROS formation induced by tert-butyl hydroperoxide (1 mM) in normal human colon fibroblast cell lines (CCD-18Co) and mouse macrophage cells (RAW264.7) when pre-treated with concentrations 500–1000 and 100–500 µg/mL of the digests, respectively. The bioactive compounds of the yogurt with grape skins obtained by digestion reduced NO production in LPS-induced RAW264.7 macrophages, showing anti-inflammatory potential. To the best of our knowledge, this is the only study aimed at evaluating the potential benefits of grape skins when used to formulate food, and it opens new perspectives for further studies to understand how they can be used to enrich snacks to increase their antidiabetic potential [107].

Concerning metabolic diseases, a study carried out by Doshi et al. [108] showed that grape skins may be a new source of insulin secretagogues. This result suggests its possible application in type II diabetes treatment [108]. The potential efficacy of grape skin in the prevention and therapy of obesity-related metabolic syndrome was observed in vivo when co-administered with HFD to male mice. Grape skin extract mediated insulin sensitivity and glucose homeostasis, attenuated oxidative stress by lowering the MDA and carbonyl levels in the muscle and adipose tissues, and mitigated inflammatory markers (TNF-α and IL-6) [109].

### 3.3. Seeds

Grape seeds’ effects on gut microbiota modulation and intestinal health were also accessed by in vivo studies. Oral administration of grape seed extract mediated parameters had a positive short-term consequence on the variations in the microbiota in female Wistar rats, which was accompanied by a reduction in the ratio of *Firmicutes*-to-*Bacteroidetes* in the gut and augmentation of the plasma glucagon-like-peptide-1 level that was allegedly attributed to the harmonious execution of metabolic processes involved in metabolic disorders [110] (Figure 3, Table 2). Twelve weeks of oral supplementation in mice with grape seed, 140 or 160 mg/kg/day, decreased the intestinal permeability and increased the fecal total antioxidant capacity and serum TNF-α levels compared with the control [111]. Moreover, grape seed extract reduced the number of proliferating nuclear antigen-positive cells (PCNA) per crypt and downregulated the mitogen-activated protein kinase’s growth signaling, evidenced by the reduced phosphorylation of the extracellular signal-regulated kinases 1 and 2 in the colon [111] (Figure 3, Table 2). In another in vivo study, IL-10-deficient and C57BL/6 wild type female mice were used to test the effects of the oral water supplementation with grape seeds at 0.1% w/w on ileal inflammation [112]. Grape seed decreased the intestinal crypt depth and increased the villus vs. crypt length ratio in the terminal ileum of IL-10-deficient mice [112]. In this IL10-deficient mice model, the oral grape seed administration also decreased the proliferation and enhanced the differentiation of the intestinal epithelial cells, suppressing the NF-κb [112] (Figure 3, Table 2). Moreover, grape seed administration reduced intestinal cell autophagy by decreasing the expression of beclin-1 (Bcl-1) when compared against C57BL/6 wild type mice as a control [112]. GP seeds, at doses of 0.05, 0.1, and 0.15 mg, have a protector effect against ulcerative colitis induced in laboratory mice by acetic acid, as observed by histological examination (Figure 3, Table 2). Colon sections of the treated animals revealed the absence of epithelial lesions and the presence of a few rare inflammatory cells in the mucosa by the 7th day of the treatments, indicating the inhibition of the colonic inflammation induced by acetic acid [113]. These results are following the ones reported above for GP, i.e., seeds also produced modulation of antioxidant status and inflammation. But these results also show that seeds, and probably other wine by-products, can also be important in cancer prevention and treatment. The decrease in PCNA-positive cells in intestinal crypts promoted by grape seeds can reveal their potential to inhibit carcinogenesis by suppressing uncontrolled neoplastic cell proliferation. Moreover, the downregulation of Bcl-1 expression by grape seeds indicates that this wine by-product can effectively induce autophagy, which is a process that eliminates cancer cells. In conclusion, grape seeds can be used in the prevention and/or treatment of intestinal carcinogenesis through the induction of autophagy.

The cardioprotective effects of grape seeds were explored in vivo, by inducing cardiotoxicity in rats with cyclophosphamide (a single dose of 200 mg/kg/body weight). The pretreatment with grape seed extracts (oral administration on rats, 150 and 300 mg/kg doses for 6 weeks) protected the liver and heart tissue and improved oxidative and apoptotic biomarkers, as well as the activity of liver and heart function enzymes [114]. A clinical trial was recently carried out to evaluate the effect of grape seed proanthocyanidin extract (400 mg) on blood pressure and vascular endothelial function in middle-aged Japanese adults with prehypertension. The results revealed an improvement in vascular elasticity and a decrease in SBP by 13 mmHg after 12 weeks [115] (Figure 3, Table 2).

An in vitro study carried out by Antonella Leone and coauthors [116] showed that grape seed extracts induce apoptotic cell death in MCF-7 breast cancer cells, which is mediated by improving gap-junction-mediated cell-cell communications through reallocating connexin-43 (cx43) proteins on plasma membranes and controlling cx43 mRNA expression [116]. Considering this result and the above-mentioned potential to suppress carcinogenesis via modulation of antioxidant status and induction of autophagy, further research should focus on grape seeds’ anti-cancer effects.

In the presence of grape seed, skin, and steam extracts in experimental trials on mice showed that, in the pancreatic islets, there is a 2- to 8-fold increase in insulin secretion at a concentration of 5.5 mM and 16.5 mM glucose, which suggests an antidiabetic protection [108]. The first studies carried out to evaluate the health effects of grape seeds were conducted with the oil extracted from them. The results give important insights into the mechanisms by which grape seed oil can help prevent metabolic disorders. One of the first studies was carried out by Irandoost, Ebrahimi-Mameghan, and Pirouzpanah (2013) [117] and was conducted to assess the effect of grape seed oil on inflammation and insulin resistance in overweight and/or obese adult females. They observed that consumption led to an improvement in inflammation and insulin resistance in overweight and/or obese women possibly because of tocotrienols and phenolic components. Muscadine grape seed oil ameliorates adipocyte inflammation by decreasing mRNA levels of IL-6, IL-8, and MCP-1 in human adipose stem cells [118]. Virgin grape seed oil was tested in vivo in mice fed an HFD, and it was observed that it has the potential to alleviate insulin resistance and improve its energy metabolism disorder by increasing respiratory exchange rate and energy consumption [119]. Regarding insulin resistance, it was speculated that grape seed oil has a protective effect on hexokinase and alpha-glucosidase activities and improves leptin resistance [119]. Moreover, grape seed oil has considerable hypolipidemic effects and could be used in the prevention and treatment of hyperlipidemia and, consequently, atherosclerosis and cardiovascular disease [119]. A recent clinical trial established that the consumption of grape seed oil (10 mL/d) is beneficial to reducing body weight, fasting glucose, and low-density lipoprotein (LDL) in individuals with familial hypercholesterolemia and overweight [120]. Some studies have also compared the effects of grape seed oils and other vegetable oils. Wall-Medrano et al. [121] carried out an in vivo experiment to compare the lipids and antioxidant profile of grape seed oil, corn oil, grape seed oil, and coconut oil in healthy Wistar rats under a sub-chronic intake (28 days). They observed that rats fed with grape seed oil and corn oil had a similar deposition of total fats in the liver, which was lower than the group fed with coconut oil. According to the study results, this grape seed oil effect could be related to more efficient reverse-cholesterol transport, rather than antioxidant reaction or hepatic phytosterol deposition. Comparisons between vegetable oil were also performed by clinical trial. Ebrahimi-Mameghani, Irandoost, and Pourmoradian (2019) [122] compare the effects of grape seeds oil and sunflower oil consumption on inflammation in overweight or obese women. They observed that both oils improved lipid profiles in overweight and obese women, but only the changes in the LDL and high-density lipoprotein were different between the grape seed oil and sunflower oil groups. Grape seed oil effects were also compared with olive oil ones in a clinical trial with patients with hyperlipidemia. The results showed that patients of the grape seed oil group presented lower total triglyceride levels; however, SBP was significantly decreased by olive oil intervention [123]. These studies suggest that grape seed oil can be potentially applied to control certain inflammatory conditions and metabolic diseases. As for other wine by-products, animal studies demonstrated the effectiveness of grape seeds in glycemic control and insulin sensitivity. The higher number of clinical trials carried out with grape seed oil show that this wine by-product may represent preventive strategies that can be considered in patients with pre-diabetic conditions such as obesity, hypertension, impaired fasting glucose, metabolic syndrome, family history of diabetes, and congestive heart failure.

Grape seed oil has also cardioprotective effects. In a preliminary test over 14 days performed on rats, pretreatment with 4 mL/kg/day of grape seed oil was applied, following the experimental induction of ischemia by a single administration of isoproterenol (ISO) 45 mg/kg. Ventricular conduction was decreased remarkably by grape seed oil pretreatment. Both, levels of proinflammatory cytokines and the myocardial fraction of creatine kinase were reduced by grape seed oil, which provides a cardioprotective effect in ISO-induced myocardial ischemia [124] (Figure 3, Table 2).

The anti-inflammatory and antioxidant effects of grape seed oil were also shown, both in vitro and in vivo, for other health conditions. Ismail, Salem, and Eassawy (2015, 2016) [125,126] carried out in vivo studies to evaluate its protective effects against carbon tetrachloride-induced damage to the liver and brain in γ-irradiated rats. Regarding the liver, the authors suggested that grape seed oil exhibited a protective effect by downregulating the expression of IL-6, TNF-α, and NF-κB [126] (Figure 3, Table 2). In vitro studies were also carried out with the unsaponifiable fraction of grape seed oil in LPS-treated human primary monocytes and murine macrophage cell line RAW 264.7i, and the antioxidant effects observed were attributed to the suppression of the intracellular production of ROS and nitrite levels as a consequence of reduced NO synthase 2 gene expression [127]. Due to its anti-inflammatory and antioxidant effects, recent in vivo studies show a promising role for grape seed oil in the prevention and/or treatment of cadmium-induced nephrotoxicity [128], osteoarthritis [129], and ulcerative colitis [130].

Based on the results of an in vivo study, grape seed oil could also improve Alzheimer’s disease, since it had remarkable cognitive-enhancing activity by preventing the deleterious effect of Scop, especially on acetylcholine levels in male rats [131].

### 3.4. Leaves

Few studies were carried out with grapevine leaves to understand their potential to prevent or treat some diseases. Dani et al. [132] analyzed organic and conventional extracts from grapevine leaves in vivo for their neuroprotective effects. The authors tested leaf extracts’ capacity to reduce protein and lipid damage and their enzymatic antioxidant activity. The results confirmed that organic extracts have a protective effect on oxidative deterioration (caused by hydrogen peroxide in the brain of rats) of lipids and proteins in the hippocampus and cerebellum tissues. The conventional extracts reduced thiobarbituric acid reactive species levels in the cortex [132].

Recently, the effect of the intragastric application of leaf extract (400 mg (kg^−1^ day^−1^) in mice fed with HFD, containing 60% kcal from fat was studied. The leaf extract inhibited pancreatic lipase secretion, supported fibroblast growth factor-15 secretion (which stops the synthesis of bile acids and fatty acids), and reduced food intake by suppressing orexigenic neuropeptide-Y. These changes promoted a decrease in serum cholesterol and LDL in triglycerides and the amount of tissue fat (Figure 3, Table 2). As observed for other wine by-products leaf extracts have anti-obesity effects [133].

### 3.5. Stems

The grapevine stem compounds, ampelopsin A and piceatannol, have the potential to inhibit amyloid-protein 25–35 (Aβ) [134,135]. In vivo ampelopsin A protected against brain cell dysfunction by blocking the aggregation of Aβ [134]. Regarding piacetamol (a hydroxyresveratrol), besides decreasing neuronal inflammation in microglial cells, it also presents cardioprotective activity [136].

Quero et al. [137] explored the effects of grape stem extracts on cancer cells (Caco-2, MCF-7, and MDA-MB-231) and on intestinal barrier cells (differentiated Caco-2 cells), and suggested that grape stem extracts could have anti-cancer and antioxidant activities. Grape stem extracts promoted cancer cell apoptosis. In Caco-2 cells, the extract exerts an inhibitory effect on the antioxidant enzyme thioredoxin reductase 1, and therefore a decrease in ROS production at the cellular level [137].

### 3.6. Grapevine Shoots

Grapevine shoot extracts were tested by Empl et al. [138] in the prevention of human gastrointestinal cancer. Using an ApcMin mice model, which was subject to an HFD like a human model of adenomatous polyposis, the authors observed that grapevine shoot extracts reduce the number (in males) and volume (in females) of intestinal adenoma. Moreover, the authors observed that grapevine shoot extracts also reduce the size growth and the number of APC10.1 cells derived from one ApcMin mouse. More studies are needed to confirm that grapevine shoot extracts can be used in the prevention of human gastrointestinal cancer [134].

**Table 2:** Effects on body functions of food made with wine by-products or their extracts achieved in pre-clinical and clinical trials.

By-Product	By-Product Health Benefits	Reference
Grape pomace	Microbial gut homeostasis improvement (non-beneficial bacteria inhibition and stimulation of probiotic bacteria growth)	In vivo: [89,90,91,92,93,94]
	Anti-inflammatory	In vitro: [96] In vivo: [91,97,101,102,103,104]
	Cardioprotective	In vivo: [98,99,100,101]
Skins	Antioxidant	In vitro: [107,109]
	Anti-inflammatory	In vitro: [107,109]
Seeds	Microbial gut homeostasis improvement (non-beneficial bacteria inhibition and stimulation of probiotic bacteria growth)	In vivo: [110]
	Antioxidant	In vivo: [111,114,121,125,126,128,129,130] In vitro: [127]
	Anti-inflammatory	In vivo: [111,112,113,124,125,126,128,129,130] Clinical trial: [117,122,123]
	Anti-cancer	In vivo: [111,112] In vitro: [116]
Leaves	Neuroprotective	In vivo: [132]
	Antioxidant	In vivo: [132]
Stems	Brain dysfunctions	In vivo: [134,136]
	Anti-inflammatory	In vivo: [136]
	Antioxidant	In vitro: [137]
	Anti-cancer	In vitro: [137]
Grapevine shoots	Anti-cancer	In vivo: [138] In vitro: [134]

## 4. Conclusions

Wine production generates a huge variety of residues, such as grape marc, vine shoots, stalks, pomace, and wine leaves. Wine by-products have high potential as food ingredients, since they facilitate increased sustainability increase in the wine industry by reusing a product that is usually considered waste. The taste and attractive color of wine by-products provide opportunities for the development of new foods with high nutritional value and health benefits and might even be considered natural alternatives to traditional synthetic additives. Wine by-products, therefore, present an interesting opportunity for adding value by recycling a waste stream and representing food ingredients with high contents of polyphenols. The current review of wine by-products includes management practices that lead to the diminution of their pollution load by promoting the advance of their content in valuable ingredients (e.g., antioxidants). These practices have a positive ecological and economic impact on wineries.

Since ancient times, vine roots, seeds, and leaves have been used widely as food preservatives and flavoring agents, but also as part of traditional medicine in various cultures. Their antimicrobial, antioxidant, anti-inflammatory and anticancer effects rely on their high polyphenol content. Many of these compounds are also present in wine by-products and could be used in the food industry to prevent microbial spoilage, product safety and prolonged shelf life, and potential health effects for the consumers. In this review we showed that the enrichment of food with wine industry by-products has several health benefits, which have been proven by several in vitro, in vivo, and clinical studies. Current research has demonstrated that foodstuffs produced with wine by-products or their extracts exert antioxidant, anti-inflammatory, cardioprotective, and anticancer actions in vitro and in vivo, which attest to the health benefits of their intake. Pre-clinical studies show that wine by-products have the potential to scavenge free radicals and modulate the inflammatory process. They inhibit the key inflammatory mediator, NFκB, and proinflammatory enzymes such as COX-2, MAPK, and protein kinase-C. They mostly exhibit a mitigating effect on inflammatory cytokines and increase anti-inflammatory cytokines. These foodstuffs represent enormous promise in the prevention of chronic diseases. Understanding the nature of wine by-product compounds, their combinations, and the molecular mechanism of action in eliciting bioactivity is vital. Further pre-clinical, epidemiological, and follow-up studies are thus warranted to explore the maximum nutraceutical potential of wine by-products in food applications.

Nowadays, scientists, politicians, and winemakers talk about sustainability, but the truth is that the implementation of sustainable practices is very scarce. Using wine by-products to enrich food provides an opportunity to improve economic, social, and environmental dimensions. It is urgent that the scientific community and producers look for more profitable and sustainable options. Sustainability requires the maximum utilization of all raw materials and wine by-products produced, and minimum disposal of waste flow. Modern wineries must embrace strategies of management and valorization that allow for both the recovery and recycling of valuable ingredients, and the creation of new products. The current review fills a gap in transferring knowledge between academia and industry by providing a reference for winemakers, professionals, and producers active in the field, attempting to optimize wineries’ performance and reduce their environmental impact. The major goal of this review is to inspire all relevant participants to develop real commercialized applications.

## Figures and Tables

**Table 1:** Summarized list of the most relevant phenolic compounds in the parts that make up Grape Pomace (skins, seeds, stems).

Chemical Structure	Phenolic Compounds	Grape Pomace	References
Flavonoid	Procyanidins (polymers of catechin), flavan-3-ol monomers; catechin, epicatechin and oligomers (B1, B2, B3, B4 and the trimer C1)	Seeds and skins	[70,71,72,73,74,75,76,77]
	Prodelphinidins (polymers of gallocatechin), gallocatechin, epigallocatechin, epigallocatechin gallate	Skins	[74,76,77,78]
	Anthocyanins; malvidin-3-O-glucoside, cyanidin-3-O-glucoside, delphinidin-3-O-glucoside, peonidin-3-O-glucoside, and petunidin-3-O-glucoside	Skins and pulp	[24,71,73,75,79]
	Flavonols (quercetin 3-O-glucoside, quercetin 3-glucuronide, myricetin 3-O-glucoside, kaempferol 3-O-glucoside, laricitrin 3-O-glucoside, isorhamnetin 3-O-glucoside, and syringetin3-O-glucoside)	Seeds and skins	[80,81,82]
Non-Flavonoid	Hydroxybenzoic acids (gallic acid, protocatechuic acid, vanillic acid)	Seeds, skins and stems	[83,84]
	Stilbenes (glucosides piceid, resveratrol, astringin, viniferin)	Stems, seeds skins	[73,85,86]
	Hydroxycinnamic acids (caffeic acid, caftaric acid, p-coumaric acid, fertaric acid)	Skins, seeds and stems	[85,87]
